# Effectiveness of an 8-Week Web-Based Mindfulness Virtual Community Intervention for University Students on Symptoms of Stress, Anxiety, and Depression: Randomized Controlled Trial

**DOI:** 10.2196/18595

**Published:** 2020-07-17

**Authors:** Christo El Morr, Paul Ritvo, Farah Ahmad, Rahim Moineddin, MVC Team

**Affiliations:** 1 School of Health Policy and Management York University Toronto, ON Canada; 2 School of Kinesiology and Health Science York University Toronto, ON Canada; 3 Dalla Lana School of Public Health University of Toronto Toronto, ON Canada

**Keywords:** virtual community, virtual care, mindfulness, depression, anxiety, stress, students, online, randomized controlled trial, Canada

## Abstract

**Background:**

A student mental health crisis is increasingly acknowledged and will only intensify with the COVID-19 crisis. Given accessibility of methods with demonstrated efficacy in reducing depression and anxiety (eg, mindfulness meditation and cognitive behavioral therapy [CBT]) and limitations imposed by geographic obstructions and localized expertise, web-based alternatives have become vehicles for scaled-up delivery of benefits at modest cost. Mindfulness Virtual Community (MVC), a web-based program informed by CBT constructs and featuring online videos, discussion forums, and videoconferencing, was developed to target depression, anxiety, and experiences of excess stress among university students.

**Objective:**

The aim of this study was to assess the effectiveness of an 8-week web-based mindfulness and CBT program in reducing symptoms of depression, anxiety, and stress (primary outcomes) and increasing mindfulness (secondary outcome) within a randomized controlled trial (RCT) with undergraduate students at a large Canadian university.

**Methods:**

An RCT was designed to assess undergraduate students (n=160) who were randomly allocated to a web-based guided mindfulness–CBT condition (n=80) or to a waitlist control (WLC) condition (n=80). The 8-week intervention consisted of a web-based platform comprising (1) 12 video-based modules with psychoeducation on students’ preidentified life challenges and applied mindfulness practice; (2) anonymous peer-to-peer discussion forums; and (3) anonymous, group-based, professionally guided 20-minute live videoconferences. The outcomes (depression, anxiety, stress, and mindfulness) were measured via an online survey at baseline and at 8 weeks postintervention using the Patient Health Questionnaire-9 (PHQ9), the Beck Anxiety Inventory (BAI), the Perceived Stress Scale (PSS), and the Five Facets Mindfulness Questionnaire Short Form (FFMQ-SF). Analyses employed generalized estimation equation methods with AR(1) covariance structures and were adjusted for possible covariates (gender, age, country of birth, ethnicity, English as first language, paid work, unpaid work, relationship status, physical exercise, self-rated health, and access to private mental health counseling).

**Results:**

Of the 159 students who provided T1 data, 32 were males and 125 were females with a mean age of 22.55 years. Participants in the MVC (n=79) and WLC (n=80) groups were similar in sociodemographic characteristics at T1 with the exception of gender and weekly hours of unpaid volunteer work. At postintervention follow-up, according to the adjusted comparisons, there were statistically significant between-group reductions in depression scores (β=–2.21, *P*=.01) and anxiety scores (β=–4.82, *P*=.006), and a significant increase in mindfulness scores (β=4.84, *P*=.02) compared with the WLC group. There were no statistically significant differences in perceived stress for MVC (β=.64, *P*=.48) compared with WLC.

**Conclusions:**

With the MVC intervention, there were significantly reduced depression and anxiety symptoms but no significant effect on perceived stress. Online mindfulness interventions can be effective in addressing common mental health conditions among postsecondary populations on a large scale, simultaneously reducing the current burden on traditional counseling services.

**Trial Registration:**

ISRCTN Registry ISRCTN12249616; http://www.isrctn.com/ISRCTN12249616

## Introduction

Yearly, 1 in 5 people in Canada experience a mental health problem [1,2], and young people aged 15-24 are more likely to experience mental illness than any other age group [3]. In the United States about half of the population will meet the criteria for a DSM-IV disorder sometime in their lives [4]. However, the first onset of mental disorders occurs in childhood or adolescence [5], and it is estimated that 70% of mental health challenges have their onset during that period [6].

University students are experiencing increases in psychological distress on North American campuses. In 2013, a student survey of 32 Canadian postsecondary institutions reported high anxiety (56.5%), hopelessness (54%), seriously depressed mood (37.5%), and overwhelming anger (42%) [7,8]. A similar survey in 2016 revealed even higher distress levels [8]. In 2013, a study of 997 students at York University (site of this study) indicated that 57% reported depression scores sufficient for diagnosable clinical depression, whereas 33% reported anxiety scores in ranges typically indicative of panic disorder and generalized anxiety disorder [9]. The situation appears similar at universities in the United States [10,11] and worldwide—in 2018, the World Health Organization reported increasing mental disorders in college and university students worldwide [12]. Distress during university attendance is critical to address, especially considering that 70% of all mental health problems appear before the age of 25 and, when untreated, can become long-standing and significant impairments affecting multiple life domains [6].

University student distress is both an individual and a societal challenge. Losses in productivity at work and during study due to distress and mental disorders are associated with indirect but major economic burdens [13]. Canadian estimates show that mental disorders cost US $51 billion yearly, with 9.8% due to direct medical costs; 16.6% and 18.2% due to long-term and short-term work loss, respectively; and 55.4% due to the loss of healthy function (ie, loss of the utilities of vision, hearing, speech, mobility, dexterity, emotion, cognition, and pain as assessed in the Health Utilities Index Mark 3 system) [14].

While mental distress and disorders are becoming more prevalent in students, the counseling offered in colleges and universities is not keeping pace with demand. For example, from 2007 to 2012, full-time enrollment in the Ontario college system increased from 167,000 to 210,600 (a 26% increase), whereas the number of counselors employed in the college system increased from 146 to 152.7 (a 4.6% increase) [15]. This discrepancy leaves students underserved and counselors overwhelmed amidst the increasing distress [16].

Mindfulness-based interventions have been demonstrated to positively impact psychological and physical health [17-19] with multiple meta-analyses demonstrating positive impacts in clinical and nonclinical populations [20-24]. However, with large numbers of students (50,000-60,000 on some campuses) there may not be enough trained personnel to directly convey helpful mindfulness-based practices. A recent systematic review showed the impact of online mindfulness interventions on depression, anxiety, and stress [25]; however, there is no clear indication as to which specific intervention components were specifically effective. Moreover, in the electronic health (eHealth) domain, virtual communities (VCs) [26], that is, online communities, have been used in health care to provide e-education tools and online support with the goal of empowering active participants in health enhancement [27-29]. VCs can scale up mindfulness interventions at lower costs to wider ranges of students, especially those restricted from attending clinics due to time–place discontinuities. VCs preserve anonymity (with reductions in stigmatization) while promoting voluntary supportive interpersonal connections.

We developed a web-delivered mindfulness program (Mindfulness Virtual Community [MVC]) to reduce depression, anxiety, and stress in university students and conducted a randomized controlled trial (RCT) targeting undergraduate students at a Canadian university to examine its effectiveness. The MVC contained analytics to measure the use of each included component. Following a successful pilot RCT [29], we wanted to further investigate whether symptoms of depression, anxiety, and stress would be significantly reduced when compared with waitlist controls (WLCs).

## Methods

### Trial Design and Ethical Approval

This study consisted of a 2-arm parallel-design RCT comparing the web-based MVC program with a WLC group. The Human Participant Research Committee at York University provided research ethics approval for the RCT (Certificate No.: e2016-345).

### Participants and Recruitment

Eligibility criteria were applied to recruit undergraduate students who were at least 18 years of age, reported English language fluency, self-rated high confidence of completing the study, and were actively enrolled in an undergraduate program. Their ability to use a computer and smartphone and internet literacy were assumed to be de facto skills. Students were excluded if they indicated substance abuse or episodes of psychotic behaviors during the month prior to the trial.

The study was advertised using multiple strategies including study posters, class announcements on permission of course directors, and email invitations via listservs of student associations in the Faculty of Health and Faculty of Liberal Arts. Interested students contacted the research staff via email or phone and were screened for student registration, substance abuse, and indications of psychoses. If substance abuse or psychotic behaviors “interfered in routine life within last month,” students were excluded and provided with a list of accessible mental health resources. Eligible and willing students received detailed information in-person about the study and provided informed written consent. Participants had the option to receive an honorarium of CAD 50 (US $37.5) or 2% in course grade (for professors who gave this permission) or 3 credits (equivalent to 2% course grade) in the Undergraduate Research Participation Pool of the Department of Psychology. Each participant also received a resource list that included information about health and social services on campus and in the community (eg, the 24 × 7 *Good to Talk* helpline for postsecondary students in Ontario). Our protocol included a safety mechanism whereby participants were asked verbally and on the consent form to contact the research staff if they felt distress during the trial period so that “limited counseling with a clinical psychologist could be arranged, if needed.” The collaborating psychologist was at arm’s length from the trial. No instance of such request arose during the reported study period.

A sample of 480 students (240 students per group) was recruited over 3 semesters (Fall 2017, Winter 2018, and Fall 2018). However, the 3 samples could not be combined due to substantial differences in the campus environment. Notably, in the Fall 2017 semester the platform functionalities presented connection challenges to students and the platform did not capture the user analytics correctly via the built-in tools, a problem which was corrected for subsequent semesters. In addition, during the Winter 2018 semester the university was disrupted by an employee strike of 3 months’ duration. Prior to the Fall 2018 semester (the semester during which this study was undertaken), the strike was resolved and the university resumed routine functioning. This article is based on the sample recruited in Fall 2018 (September 23, 2018, to November 18, 2018).

### Randomization

Participating students were randomized to the MVC intervention or the WLC using 1:1 block randomization. The randomized allocation sequence was computer generated by an offsite team member (RM), and allocations were concealed in sequentially numbered opaque envelopes [30]. These envelopes were opened only after written consent was obtained, ensuring that participants and research staff were blind prior to the allocation. Each participant in the MVC group received a unique ID and a temporary password; participants changed their passwords after the first login while IDs remained the same to eliminate the possibility of multiple accounts or identities. Participants in all groups completed online questionnaires at baseline (T1) and 8 weeks (T2).

### Intervention

**Figure 1:**
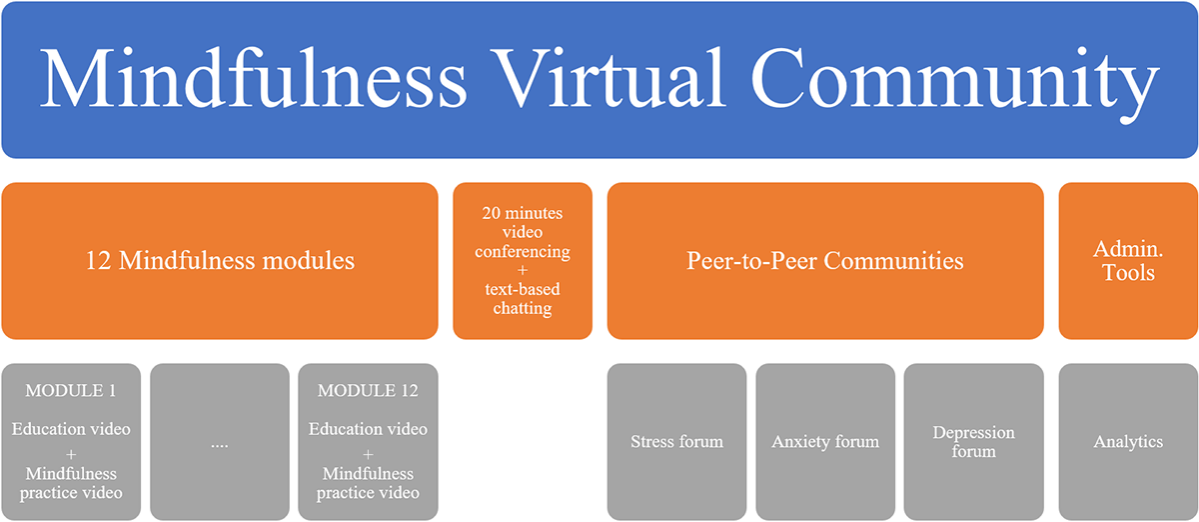
The mindfulness virtual community design.

The MVC intervention was 8 weeks in duration. The intervention comprised 3 components: (1) 12 student-specific mental health modules conveyed by online video; (2) 3 anonymous discussion boards dedicated to depression, anxiety, and stress; and (3) an anonymous 20-minute group-based live videoconference led by a moderator (a counselor with a master’s degree in psychology and training in mindfulness) during which students could raise and discuss topics covered in the modules (Figure 1).

Each of the 12 mental health modules consisted of 1 educational content video and 1 mindfulness practice video recorded in both male and female voices and offered in both high and low resolution (a total of 8 videos per module); participants could choose the type of video they wanted to watch for each module. The videos were available for participants 24 hours/day to watch or listen to on computers, phones, or tablets at their convenience. The module scripts and audio recordings were created by one of the investigators with extensive experience as a clinical psychologist and researcher in mindfulness (PR) [31-36], and they were based on mindfulness and cognitive behavioral therapy (CBT) principles and informed by the prior student-based focus group study [37,38]. The choice of moving and still images used in the creation of the videos involved collaborative work (PR, CEM, and FA). The topics of the 12 modules and the video duration (average durations of male voice and female voice videos) are presented in Table 1. The role played by the online mindfulness moderator evolved over time across a pilot study conducted in Winter 2017 [29].

Our study is currently at stage 2 of the National Institute of Health stage model [39]. It builds on a pilot study in which we tested the platform and consists of testing the MVC in a research context with research therapists/providers. As advised by Dimidjian and Segal [40], we have addressed clinician training and engagement in mindfulness based on analytics within the platform and self-report. However, practice time outside the intervention was not measured.

**Table 1:** Topics and duration of modules.

Topics	Video duration (mm:ss)	
	Education videos	Mindfulness videos
Overcoming stress, anxiety, and depression	7:09	9:00
Mindfulness and being a student	5:18	9:14
Mindfulness for better sleep	4:40	8:13
Thriving in a fast-changing world	7:23	8:23
Healthy intimacy	7:32	9:33
De-stigmatization	6:13	9:12
No more procrastination	3:42	10:48
Pain reduction and mindfulness	3:48	9:48
Healthy body image	5:44	9:54
Healthier eating	10:10	9:26
Overcoming trauma	6:01	9:43
Relationships with family and friends	7:49	8:09

New modules were periodically released during the 8-week intervention period. Following release, they remained accessible to students for the remainder of the intervention period. The videoconferences were offered biweekly in three 20-minute evening sessions (Table 5). The students in the intervention group received email reminders from the project staff prior to the release of each new module and prior to the live videoconferences. Participants were encouraged to use the platform as often as desired. Access to technology and the internet was assumed to be of no consequence to participants; indeed, a focus group study that guided MVC design revealed that 94% (n=68/72) of the students had access to a smartphone and 93% (n=67/72) had access to laptops or personal computers at home, while all participants had free internet access on campus and almost all had internet access through smartphones or laptops [38].

The MVC was developed in partnership with the industry partner ForaHealthyme Inc. and constituted a virtual environment supportive of personal mindfulness practice and related CBT self-help and mutual help interactions between participants, and between participants and the moderator. There were 2 types of users: the student and the health professional (who moderated the discussion board dialogues and led the live videoconferences). Users had to use a login and a password to gain access to the MVC.

Once logged in, each student could (1) access the video (educational and mindfulness practice) modules; (2) access 3 peer-to-peer discussion boards, one for each of the 3 mental health conditions targeted by the RCT (anxiety, stress, and depression); (3) notify the moderator about any message posted that represented a problem to the student (eg, online bullying); (4) access a calendar to book an upcoming videoconference; (5) access a virtual videoconferencing room that allowed videoconferencing (camera and microphone being turned off as default) and private text-based chatting with the moderator; and (6) a resource page with contact information for various social and health services.

Once the moderator logged in, she accessed the same options as students with the following additional features: the ability to delete any message on the discussion boards deemed potentially distressing to other students; the ability to populate the calendar with dates and times for upcoming videoconference sessions; the ability to start a videoconference session (camera turned on by default); and the ability to respond privately to incoming text messages in the virtual videoconferencing room.

The moderator had weekly supervision sessions with the team clinician (PR) to optimize responses to the videoconferences and submitted weekly written reports (without individual names) about topics raised by students and her responses to them. The content of modules and the platform structure remained unchanged for the 8-week intervention. Only the name of the university which received the research grant and the partnering IT company name appeared on the main page of the platform.

### Primary and Secondary Outcomes and Measures

The outcomes and other variables were measured by self-report questionnaires at T1 and T2. The primary RCT outcomes were depression, anxiety, and perceived stress, whereas mindfulness was measured as a secondary outcome. It was hypothesized that symptom scores for depression, anxiety, and stress at T2 would be significantly lower in the MVC group compared with those in the WLC group, and that scores for mindfulness at T2 would be significantly higher in the MVC group compared with those in the WLC group. The outcomes were measured with the following validated scales: Patient Health Questionnaire-9 (PHQ9) [41], Beck Anxiety Inventory (BAI) [42], Perceived Stress Scale (PSS) [43], and Five Facets Mindfulness Questionnaire Short Form (FFMQ-SF) [44]. Participants also completed a sociodemographic questionnaire at the T1 survey.

### Statistical Analysis and Sample Size

The sample size was calculated for 80% power and 5% type I error to detect a standardized effect size of 0.5 or larger. The required sample size was found to be 63 students in each arm. We aimed to recruit 80 participants per arm expecting an attrition rate of 20% (n=16). Descriptive statistics were used to summarize the sample characteristics. Sample *t* test (two-tailed) for continuous measures and chi-square test for categorical variables were employed to compare the intervention and control groups at baseline.

The approach to the outcome analysis was intention to treat. To test whether the intervention could reduce depression, anxiety, and stress scores and increase mindfulness scores after 8 weeks, we utilized a generalized estimating equations method with an AR(1) covariance structure. Because there were not any patterns for missing data, missing observations (10%) were assumed to be missing at random, and a completed data set was obtained by estimating the missing observations with the multiple imputation method using the Markov chain Monte Carlo technique. Two models were considered for each dependent variable. Model 1 was fitted to investigate the effect of the interventions on depression, anxiety, stress, and mindfulness scores after 8 weeks. A negative significant effect of group assignment was interpreted as evidence that symptom scores for depression, anxiety, and stress at time point 2 were significantly reduced in the MVC group compared with the WLC group, whereas a positive significant effect of group assignment was interpreted as evidence that scores for mindfulness at time point 2 were significantly increased in the MVC group compared with the WLC group. In Model 2, demographic variables including sex, age, country of birth, ethnicity, English as first language, paid work, unpaid work, relationship status, physical exercise, self-rated health, and access to private mental health counseling were added to Model 1 to adjust for potential covariates. The analyses were performed using software SPSS version 26 (IBM).

## Results

### Recruitment

**Figure 2:**
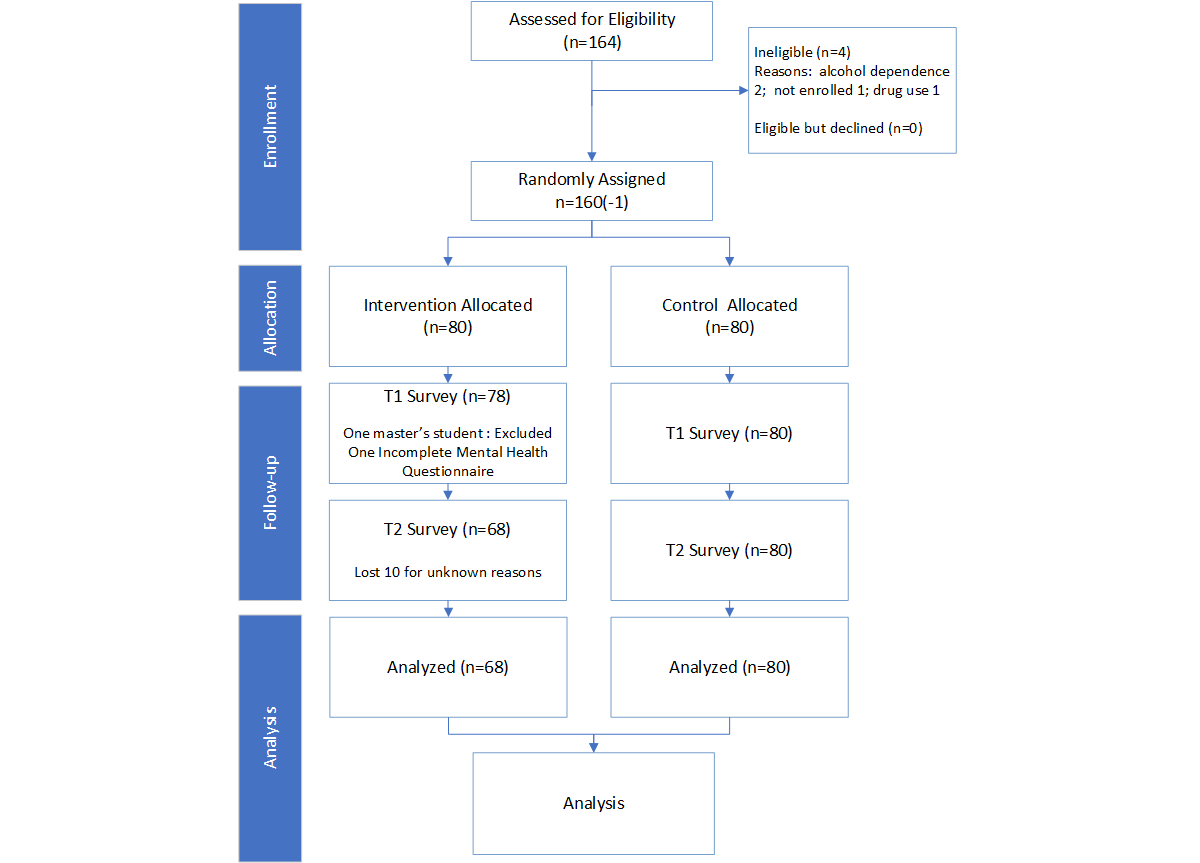
The CONSORT flow diagram for the trial.

A total of 160 undergraduate students were randomized to the MVC (n=80) or WLC (n=80) groups. One student allocated to the intervention was excluded after randomization when he clarified that he was a graduate student and had misunderstood the eligibility criteria. One additional participant in the intervention group did not complete the baseline survey for mental health instruments but completed the demographic data. Of 158 eligible students who completed the full baseline survey, the attrition rate was 6.33% at T2 (n=10; Figure 2).

### Demographics

Overall, there were 32 males (20.1%) and 125 females (78.6%) who participated with 2 students declaring *gender fluid* and *nonbinary* genders. The majority of participants were born outside of Canada (87/159, 54.7%) and reported English as their first language (93/159, 58.5%), and 20.1% (32/159) of the sample self-identified as White. The majority of participants did not have access to private mental health insurance (102/159, 64.1%). These and other characteristics were similarly distributed between the control and intervention groups (Table 2) with the exception of unpaid work, relationship, and gender: compared with the MVC group, participants in the WLC group worked on average more hours (*P*=.03), were significantly more likely (*P*=.05) to be *single with no relationship* (ie, not married nor common law, and engaged in a romantic relationship), and were significantly less likely to be *single in relationship* (*P*=.05). Finally, the proportion of females in the WLC group was significantly higher than that in the MVC group (*P*=.02).

Table 3 presents the mean and standard deviation for depression (PHQ9), anxiety (BAI), stress (PSS), and mindfulness (FFMQ-SF) scores at 2 time points. The table also provides the Cohen *d* effect sizes for mean difference in the MVC group compared with the control group at T2. The effect size shows that the levels of depression, anxiety, and stress decreased, whereas the level of mindfulness increased as a result of the intervention. There were no statistically significant differences at baseline for any of the mean scores of the 4 outcomes between the control and intervention groups. The effect sizes for PHQ9, BAI, and FFMQ-SF were between medium and large.

**Table 2:** Participant characteristics.

Characteristics		Total (N=159)	Waitlist control (n=80)	MVC Intervention (n=79)	*P* value*
Age, mean (SD); range		22.55 (6.1); 18-55	22.3 (5.9); 18-55	22.8 (6.4) 18-54	.6
**Gender, n (%)**					
	Male	32 (20.1)	10 (12.5)	22 (27.8)	**.02**
	Female	125 (78.6)	69 (86.3)	56 (70.9)	
	Gender fluid	1 (0.6)	1 (1.3)	0 (0)	
	Nonbinary	1 (0.6)	0 (0)	1 (1.3)	
**Country of birth, n (%)**					
	Canada	72 (45.3)	31 (38.8)	41 (51.9)	.11
	Other	87 (54.7)	49 (61.2)	38 (48.1)	
**First language, n (%)**					
	English	93 (58.5)	44 (55.0)	49 (62.0)	.42
	Other	66 (41.5)	36 (45.0)	30 (37.9)	
**Relationship status, n (%)**					
	Single, no relationship	102 (64.2)	58 (72.5)	44 (55.7)	.05
	Single in relationship	38 (23.9)	12 (15.0)	26 (32.9)	
	Married/common law	9 (5.7)	4 (5.0)	5 (6.3)	
	Divorced/Separated/Widowed/other	10 (6.3)	6 (7.5)	4 (5.0)	
**Ethnicity, n (%)**					
	White	32 (20.1)	15 (18.8)	17 (21.5)	.72
	Black	23 (14.5)	10 (12.5)	13 (16.5)	
	South Asian	44 (27.7)	25 (31.2)	19 (24.1)	
	Chinese	15 (9.4)	6 (7.5)	9 (11.4)	
	Other	45 (28.3)	24 (30.0)	21 (26.5)	
**Self-rated health, n (%)**					
	Poor/fair	35 (22.0)	23 (28.7)	12 (15.1)	.10
	Good	56 (35.2)	28 (35.0)	28 (35.4)	
	Very good/excellent	67 (42.1)	29 (36.3)	38 (48.1)	
**Access to private mental health, n (%)**					
	Yes	56 (35.2)	26 (32.5)	30 (37.9)	.51
	No	102 (64.1)	54 (67.5)	48 (61.7)	
**Weekly hours, mean (SD)**					
	Paid work	8.5 (9.8)	7.38 (10.3)	9.63 (9.3)	.15
	Unpaid work	2.41 (3.8)	3.06 (4.6)	1.75 (2.7)	**.03**
	Weekly physical exercise in minutes	124.5 (151.5)	108.1 (143)	140.9 (158.7)	.18

*Significant differences at *P*<.05 are highlighted in bold.

**Table 3:** Descriptive statistics for depression, anxiety, stress, and mindfulness scores.

Outcome			N		Waitlist control, mean (SD)		N		MVC intervention, mean (SD)		*P* value*		Effect size
**PHQ^a^>9-item**													
	T1	80		9.91 (6.22)		78		8.36 (5.62)		.10			
	T2	80		11.21 (6.72)		68		7.04 (5.03)		**<.001**		–0.69	
**BAI^b^21-item**													
	T1	80		17.56 (12.17)		78		14.94 (11.84)		.17			
	T2	80		18.19 (13.18)		68		10.06 (7.80)		**<.001**		–0.74	
**PSS^c^10-item**													
	T1	80		22.01 (5.32)		78		20.62 (5.91)		.12			
	T2	80		21.16 (5.01)		67		20.10 (3.85)		.16		–0.23	
**FFMQ-SF^d^24-item**													
	T1	80		70.54 (13.79)		79		71.59 (14.57)		.64			
	T2	80		67.50 (14.71)		70		74.20 (13.78)		**.005**		0.47	

*Significant differences at *P*<.05 are highlighted in bold.

^a^PHQ: Patient Health Questionnaire.

^b^BAI: Beck Anxiety Inventory.

^c^PSS: Perceived Stress Scale.

^d^FFMQ-SF: Five Facets Mindfulness Questionnaire Short Form.

### Guided Web-Based Mindfulness Versus Waitlist Control

Results from Model 1 (unadjusted) and Model 2 (adjusted) are presented in Table 4. Comparing depression in the MVC group with the WLC group, there was a statistically significant score reduction for PHQ9 at T2 in both the unadjusted (β=–2.13, *P*=.016) and adjusted analyses (β=–2.21, *P*=.01). In relation to anxiety in the MVC group compared with the WLC group, there was a statistically significant reduction in BAI score at T2 in both the unadjusted (β=–4.89, *P*=.004) and adjusted (β=–4.82, *P*=.006) analyses. Compared with the WLC group, the MVC intervention had no statistically significant effect on PSS stress scores in either the unadjusted (β=.66, *P*=.46) or the adjusted (β=.64, *P*=.48) analysis. In relation to mindfulness in the MVC group compared with the WLC group, there was a statistically significant score reduction for FFMQ-SF at T2 in both the unadjusted (β=5.94, *P*=.004) and adjusted (β=4.84, *P*=.02) analyses.

Among all the other covariates, country of birth and self-rated overall health had significant effects on depression, anxiety, and mindfulness. The variables of ethnicity, English as first language, and age had significant effects on mindfulness only.

At T2, those participants who were born outside of Canada had 2.96 units (β=2.96, *P*=.02) higher depression, 4.91 units higher anxiety (β=4.91, *P*=.02), and 5.89 units lower mindfulness (β=–5.89, *P*=.03) compared with those born in Canada.

Participants with *poor* self-rated overall health reported 3.37 units (β=3.37, *P*=.001) higher depression, 7.75 units (β=7.75, *P*<.001) higher anxiety, and –10.94 units (β=–10.94, *P*<.001) lower mindfulness than participants with *very good* self-rated overall health.

Ethnicity was divided into 5 categories: White, South Asian, Chinese, Black, and Other. At T2, compared with students of white ethnicity, students with ethnicity *other* experienced about –6.56 units lower mindfulness (β=–6.56, *P*=.04). Moreover, students whose first language was English experienced 5.97 units (β=5.97, *P*=.01) higher mindfulness than students whose first language was not English. In addition, for every year increase in age, mindfulness rose by 0.54 units (β=.54, *P*=.01).

**Table 4:** Generalized estimation equation with multiple imputation for depression, anxiety, stress, and mindfulness scales (Model 1: unadjusted and Model 2: adjusted).

Primary outcome		Unadjusted (standard error)^a^, 95% CI	*P* value*	Adjusted^b^ (standard error)^a^, 95% CI	*P* value*
**PHQ^c^9-item**					
	Intercept	9.91 (0.69), 8.56 to 11.27	**<.001**	9.62 (3.02), 3.67 to 15.57	**.002**
	Time	1.30 (0.59), 0.13 to 2.47	**.03**	1.30 (0.60), 0.06 to 2.54	**.03**
	Group	–1.51 (0.93), –3.34 to 0.32	.10	–1.01 (0.88), –2.74 to 0.72	.25
	Time × Group	–2.13 (0.89), –3.87 to –0.39	**.02**	–2.21 (0.872), –3.92 to –0.50	**.01**
**BAI^d^21-item**					
	Intercept	17.56 (1.35), 14.91 to 20.2	**<.001**	14.81 (5.16), 4.68 to 24.94	.**004**
	Time	0.63 (1.21), –1.75 to 3.00	.61	0.63 (1.21), –1.74 to 3.0	.60
	Group	–2.63 (1.89), –6.33 to 1.07	.16	–1.92 (1.75), –5.35 to 1.52	.27
	Time × Group	–4.89 (1.72), –8.25 to –1.52	**.004**	–4.82 (1.75), –8.25 to –1.39	**.006**
**PSS^e^10-item**					
	Intercept	22.01 (0.59), 20.85 to 23.17	**<.001**	20.16 (2.42), 15.35 to 24.97	**<.001**
	Time	–0.85 (.57), –1.97 to 0.26	.14	–0.85 (0.57), –1.95 to 0.25	.14
	Group	–1.39 (0.87), –3.11 to 0.32	.11	–1.15 (0.90), –2.92 to 0.62	.20
	Time × Group	0.66 (0.89), –1.09 to 2.40	.46	0.64 (0.91), –1.15 to 2.43	.48
**FFMQ-SF^f^**					
	Intercept	70.54 (1.53), 67.53 to 73.54	**<.001**	62.97 (6.92), 49.40 to 76.53	**<.001**
	Time	–3.39 (1.32), –5.98 to –0.80	.61	–3.34 (1.29), –5.86 to –0.81	**.01**
	Group	0.68 (2.24), –3.71 to 5.1	.16	–1.12 (2.07), –5.18 to 2.94	.59
	Time × Group	5.94 (2.03), 1.96 to 9.23	**.004**	4.84 (2.04), 0.81 to 8.87	**.02**

*P values <.05 are considered significant (shown with bold).

^a^Standard error of the mean score difference.

^b^Adjusted for gender, age, country of birth, ethnicity, English as first language, paid work, unpaid work, relationship status, physical exercise, self-rated health, and access to private mental health counseling.

^c^PHQ: Patient Health Questionnaire.

^d^BAI: Beck Anxiety Inventory.

^e^PSS: Perceived Stress Scale.

^f^FFMQ-SF: Five Facets Mindfulness Questionnaire Short Form.

### Platform Use

In the postintervention survey, participants reported the number of videos they had used and the frequency of use. Analyses demonstrated that participants reported watching a mean of 6 educational videos and 6 mindfulness videos per week. During the 8 weeks, the median reported watching times were 44.03 minutes for educational videos and 74.26 minutes for mindfulness videos, and the range was 0-301.93 minutes for educational videos and 0-445.53 minutes for mindfulness videos, which corresponds to 0-37.74 minutes for educational videos and 0-55.69 minutes for mindfulness videos, per week.

The survey also showed that 54% of the students (n=36/67) completed at least 50% of the videos, while the analytics showed that students logged on to the platform 444 times.

There was no statistically significant difference between the mean minutes used for listening to male voice videos versus female voice videos (t_156_=–1.2, *P*=.91).

Videoconferencing sessions were run twice a week on Wednesdays and Fridays, 3 sessions each day, at 8:00 PM, 8:30 PM, and 9:00 PM (except for the first week where the sessions were held on Tuesday and Wednesday at 9:00 PM, 9:30 PM, and 10:00 PM due to moderator scheduling issues). The number of participants in each session is shown in Table 5.

The session from 8:00 to 8:30 PM was the most preferable; on average 2.25 students attended the first session (8:00-8:30 PM), 1.3 students attended the second sessions (8:30-9:00 PM), and 2.4 students attended the third session (9:00-9:30 PM). Overall, the number of participants who attended the videoconferencing sessions was low; on average, there were 1.92 participants per videoconferencing session. In the first 4 weeks, 60 participants attended the videoconferencing sessions, whereas in the last 4 weeks the number of attendees dropped to 32.

**Table 5:** Videoconferencing session attendance.

Week and session 1		Session 2		Session 3
**1**				
	5	3	0	
	4	1	0	
**2**				
	6	2	3	
	3	1	8	
**3**				
	2	0	2	
	2	1	5	
**4**				
	2	0	1	
	4	4	1	
**5**				
	1	2	1	
	1	2	1	
**6**				
	0	0	0	
	2	0	4	
**7**				
	0	0	2	
	2	1	4	
**8**				
	0	0	3	
	2	1	3	
**Total**				
	36	18	38	

## Discussion

### Principal Results

The study investigated the effectiveness of MVC, an internet-based mindfulness–CBT environment designed to reduce symptoms of anxiety, depression, and stress in undergraduate students. The MVC intervention comprised brief online video-based modules with psychoeducational content and mindfulness practice content, peer-to-peer anonymous and asynchronous discussion forums, and 20-minute synchronous videoconferencing related to mindfulness practice with a moderator.

On testing, the MVC intervention significantly reduced depression scores (PHQ9) and anxiety scores (BAI), and significantly increased mindfulness (FFMQ-SF) compared with waitlist controls at 8 weeks. The mean depression scores of participants declined from the high end of mild depression to the low end. The intervention had no effect on stress levels (PSS), although a previous pilot MVC study showed significant reductions in stress levels in a similar population [29]. One reason for the absent effect could be higher baseline stress scores for this sample than in the pilot study (eg, 20.6 vs 19.2), which was possibly a consequence of several months of a strike that our sample experienced prior to start of Fall 2018.

In this study, the results show medium to high effect sizes for depression (–0.69) and anxiety (–0.74). This is within the higher range found in 3 studies of internet-based mindfulness [45-47], which showed significant effects on depressive symptoms at postintervention compared with control, and in which the between-group effect sizes ranged between 0.41 and 0.84. However, our study is somewhat differentiated by the mindfulness psychoeducation and practice modules being integrated with CBT principles.

Our analysis showed that students born outside of Canada and those who reported *poor* self-rated overall health had significantly higher depression and anxiety and lower mindfulness compared with students born in Canada and students with *very good* self-rated overall health. This finding aligns with reports that suggest immigrants enter the country with better mental health (than the Canadian-born population) but confront higher risks for deteriorating health after arrival [48]. Given that in Canada both population and economic growth are driven by immigration, there is a need to develop mental health services to assist new immigrants (a need recognized by the Mental Health Commission of Canada [49]). Furthermore, the link between *poor* overall health and mental health is confirmed by existing literature, as people living with chronic physical health conditions experience depression and anxiety at two times the rate of the general population [50]. Both country of birth and poor health are important factors to consider in future solutions addressing depression, anxiety, and mindfulness on Canadian campuses. Future programs might involve adaptive cultural and racialized components (eg, language, symbols) while emphasizing physical health promotion.

As our analysis also showed that students whose first language was not English experienced lower mindfulness scores than those whose first language was English, a focus on addressing language barriers and the use of multilingual mindfulness interventions might be useful.

The mean minutes for the use of videos (4.93 minutes for educational videos and 7.16 minutes for mindfulness videos) reported by participants also support the results of increases in self-rated mindfulness (Five Facet Mindfulness Questionnaire), mood improvements, and anxiety reductions.

We did not measure the effect of the intervention beyond 8 weeks in the intervention group. Future research with long-term follow-up is essential to examine long-term changes. The videoconferencing sessions were expected to accommodate all students allocated to the intervention (ie, an average of 14 students per session for six 20-minute sessions a week). Nonetheless, most of the participants did not attend the videoconferencing given the low mean attendance. These findings indicate that students were mostly interested in self-learning and self-practice, which might be partially explained by anxieties about the possible privacy loss in conferencing sessions as expressed by students during the focus group discussions held prior to the RCT [37].

It is possible that MVC participation without any related social activity is more attractive to students and, given the reported results, nonetheless effective. It seems that the application of mindfulness practice requires no necessary social contact. Mindfulness practice without social exposures might be a feature that requires further study, especially as mindfulness itself is a promising technique for cultivating self-management [51]. Other studies on self-management of chronic conditions have shown positive health outcomes such as increased autonomy and improved care quality [52]. Students were engaged in videoconferencing in the first 4 weeks (60 attendees) compared with the last 4 weeks of the intervention (32 attendees). This can be explained by the decreased time availability near the end of the semester. The lack of interest in discussion forums may indicate that forums are redundant for youths when they are already involved in multiple social media messaging platforms.

### Comparison With Prior Work

A recent systematic review on the effect of online mindfulness interventions [25] provides evidence that online mindfulness interventions significantly decrease symptoms of stress, depression, and anxiety; however, large sample studies are needed to have conclusive results. Indeed, 5 of the 10 reviewed studies had a sample larger than 100 participants [46,47,53-55]. Of the remaining 5 studies, 4 [45,56-58] had samples of between 50 and 100 participants, and 1 [59] had a sample of fewer than 50. The per-arm sample sizes were relatively small in 2 of the studies [58,59], where participants allocated to each arm were fewer than 30. Compared with these studies, our sample size was large (n=160). The findings of this study contribute evidence on the effectiveness of online mindfulness–CBT interventions in addressing the mental health challenges of undergraduate students. The existence of some objective analytics to analyze the use of the different components of the online platform is unique and will allow us to understand which components of the delivered program were most effectively used.

Our analysis points to the fact that students enrolled in our study were not interested in online discussion forums but were interested in connecting with a moderator via videoconferencing and in self-management of their symptoms using the online videos. This is important given that self-management of mental health conditions has the potential to be widely effective, while evidence of its effectiveness is modest [60]. Our work informs the rapidly evolving field of mental health self-management [61-63].

### Strengths and Limitations

This is the one of the first clinical trials of an online interactive mindfulness–CBT program for undergraduate university students in Canada and is the first to include objective background analytics. The results show that discussion forums were never used, as students were more interested in self-management and connecting with a professional via videoconferencing. This is an important finding for the design of online mental health interventions. The self-rated data and analytics enabled us to analyze video use. Another strength of the study is that it involves testing the MVC in a research context with research therapists or providers, representing stage 2 of the National Institute of Health stage model [39], a necessary step toward stage 3 efficacy trials conducted in community settings using community providers [40].

A limitation of the study is that although participants were blind to the intervention and control conditions until they opened the allocation envelopes (after consenting), this was not a single-blinded trial. Another limitation is that the study measures the effect of the program over 8 weeks, while tests of longer-term effects (over 6 or 12 months) remain to be undertaken. A further limitation is the high female preponderance in both the control and intervention groups; however, this is in line with other studies that showed the same predisposition [64,65]. Future research projects might need to address gender distribution within the sampling scheme through stratification. Besides, our research is on one site only. Future research with larger samples of participants from multiple universities and colleges would better test the generalizability of results. Missing data in the intervention group are also a limitation, but we mitigated the missing data by multiple imputation, a robust high-quality imputation method [66,67].

We also did not measure the participant’s mindfulness practice outside the platform, and hence we did not control for that analytic dimension.

Further, prior to the study, we did not verify with participants that the videoconferencing session times were aligned with their schedules, and we did not ask about these fits in the poststudy questionnaire. Such an alignment is important for the successful attendance of the videoconferencing sessions. Finally, the implementation was limited to one site.

### Conclusions

Our results suggest that an 8-week-long online mindfulness–CBT video-based program is an effective intervention for undergraduate university students in reducing symptoms of depression and anxiety. Our findings also suggest that online mindfulness interventions offer an opportunity to address common mental health conditions among postsecondary populations on a large scale, simultaneously reducing the current burden on traditional counseling services.

## Abbreviations

BAI: Beck Anxiety Inventory
CBT: cognitive behavioral therapy
FFQM-SF: Five Facets Mindfulness Questionnaire Short Form
MVC: Mindfulness Virtual Community
PHQ-9: Patient Health Questionnaire-9
PSS: Perceived Stress Scale
RCT: randomized controlled trial
WLC: waitlist control
