# Correction: Effectiveness of an 8-Week Web-Based Mindfulness Virtual Community Intervention for University Students on Symptoms of Stress, Anxiety, and Depression: Randomized Controlled Trial

**DOI:** 10.2196/24131

**Published:** 2020-09-30

**Authors:** Christo El Morr, Paul Ritvo, Farah Ahmad, Rahim Moineddin, MVC Team

**Affiliations:** 1 School of Health Policy and Management York University Toronto, ON Canada; 2 School of Kinesiology and Health Science York University Toronto, ON Canada; 3 Dalla Lana School of Public Health University of Toronto Toronto, ON Canada

In “Effectiveness of an 8-Week Web-Based Mindfulness Virtual Community Intervention for University Students on Symptoms of Stress, Anxiety, and Depression: Randomized Controlled Trial” (JMIR Ment Health 2020;7(7):e18595) the authors noted two errors.

Member names of the group author “MVC Team” were not added to the PubMed listing of this article at the time of publishing. All member names have now been added as collaborators on the PubMed listing, as follows:

Sahir Abbas, Yvonne Bohr, Manuela Ferrari, Wai Lun Alan Fung, Louise Hartley, Amin Mawani, Kwame McKenzie, Jan E Odai.

As well, MVC Team was incorrectly noted as having contributed equally. This note has been removed from MVC Team. Only Christo El Morr, Paul Ritvo, and Farah Ahmad contributed equally.

The correction will appear in the online version of the paper on the JMIR Publications website on September 30, 2020, together with the publication of this correction notice. Because this was made after submission to PubMed, PubMed Central, and other full-text repositories, the corrected article has also been resubmitted to those repositories.

